# Factors associated with suicidal ideation among university students**^1^**

**DOI:** 10.1590/1518-8345.1592.2878

**Published:** 2017-05-15

**Authors:** Hugo Gedeon Barros dos Santos, Samira Reschetti Marcon, Mariano Martínez Espinosa, Makilin Nunes Baptista, Paula Mirianh Cabral de Paulo

**Affiliations:** 2 MSc, RN, Unidade de Atenção Psicossocial, Hospital Univeristário Julio Muller, Cuiabá, MT, Brazil.; 3 PhD, Adjunct Professor, Faculdade de Enfermagem, Universidade Federal de Mato Grosso, Cuiabá, MT, Brazil.; 4 PhD, Adjunct Professor, Departamento de Estatística, Universidade Federal de Mato Grosso, Cuiabá, MT, Brazil.; 5 PhD, Professor, Departamento de Psicologia, Universidade São Francisco, Itatiba, SP, Brazil.; 6 Undergraduate student in Nursing, Universidade Federal de Mato Grosso, Cuiabá, MT, Brazil. Scholarship holder at Conselho Nacional de Desenvolvimento Cientifico e Tecnológico (CNPq), Brazil.

**Keywords:** Suicidal Ideation, Univesities, Students, Adolescent, Risk Factors

## Abstract

**Objective:**

to analyze the factors associated with suicidal ideation in a representative sample of university students.

**Methods:**

cross-sectional study, carried out with 637 students of the Federal University of Mato Grosso. The presence of suicidal ideation, demographic and socioeconomic variables, use of alcohol through the Alcohol, Smoking and Substance Involvement Screening Test, and depressive symptoms (Major Depression Inventory) were investigated. Bivariate analysis was performed with the Chi-square test and multivariate analysis using the Poisson regression model.

**Results:**

it was found that 9.9% of the students had suicidal thoughts in the previous 30 days and, in the bivariate analysis, the variables economic class, sexual orientation, religious practice, suicide attempts in the family and among friends, alcohol consumption and depressive symptoms were associated with suicidal ideation. In the multivariate analysis sexual orientation, suicide attempts in the family and the presence of depressive symptoms remained as associated factors.

**Conclusion:**

these findings constitute a situational diagnosis that enables the formulation of academic policies and preventive actions to confront this situation on the university campus.

## Introduction 

Suicidal ideation is a key element in a process called suicidal behavior, and emerges as a trigger for other components, i.e. the suicide attempt, and committing suicide^1^. In university students suicidal ideation may present at a particularly important moment, due to leaving adolescence and entering the young adult age and/or the adversities experienced in academic life^2^.

Suicide is identified as the second leading cause of death among university students, second only to self-inflicted injuries^3^. According to the World Health Organization (WHO), in 2012 it was estimated that 804,000 people committed suicide in the world. Among young people (aged 15 to 29 years), an increase in cases has been shown, accounting for 8.5% of deaths in this age group worldwide^4^. Evidence of the growth in this population segment is of concern, considering the possibility of years to be lived, productivity and transformation in the lives of these young people who are entering the academic world^3^. 

A report developed with 105,000 university students of the United States of America (USA) regarding suicidal behavior, showed that 3.7% had thought about suicide in the previous 12 months and 1.5% in the previous two weeks. Regarding suicide attempts, the report highlighted that 0.8% of the students had attempted suicide in the previous 12 months, 0.3% in the previous two weeks and 0.2% in the previous days^5^.

A study conducted with 258 Colombian university students, showed that 31% presented suicidal ideation^6^. Furthermore, a study conducted in northeastern Brazil showed, among 637 university students, a prevalence of 7.5% for suicide attempts and 52.5% for suicidal ideation^7^.

Diverse factors have been suggested in the literature as being associated with suicidal ideation, which shows that this is a multifactorial or multidimensional event^1^ ^,^ ^8^. More subjective aspects such as hopelessness, impulsivity, aggression, body perception, communication difficulties and lack of social belonging have been suggested as possible factors that trigger the suicidal ideation process^8^ ^-^ ^10^. Other aspects, such as demographic and socioeconomic variables, sexual orientation, religious practice, suicidal behavior in the family and among friends, alcohol consumption and depressive symptoms have also been shown in the literature to be relevant^6^ ^-^ ^8^ ^,^ ^11^ ^-^ ^13^.

Therefore, among university students, the different possible factors associated with suicidal ideation may be present at an unusual time of life when many changes are taking place, which include challenges of the personal, social and academic development process that requires maturity and autonomy to take decisions considering the strict determinations of the academic environment^11^ ^,^ ^14^ ^-^ ^15^.

Thus, identifying the factors that are associated with suicidal ideation in university students can be an important tool for the planning of prevention and protection activities, both by university managers, as well as the health teams who assist these students on and off campus. The international literature has produced some information about suicidal ideation directed toward this population^1^ ^,^ ^6^ ^,^ ^11^ ^,^ ^14^ ^-^ ^15^, however, there is a lack of national studies on this subject in the university setting, a situation that reinforces the need for studies with this population. 

Based on this, the aim of this study was to analyze the association of demographic and socioeconomic factors, suicidal behavior in the family and among friends, alcohol consumption and depressive symptoms with suicidal ideation among university students.

## Method 

This cross-sectional study was conducted with students of the Federal University of Mato Grosso-UFMT, Brazil. The sample selection method was random cluster (classes) and stratified (large areas) sampling, in which all groups were equally likely to be drawn. To determine the size of the sample, a confidence level of 95%, a ratio of 50% and an estimation error of 3.5% were considered, totaling 714 students eligible for the study.

The inclusion criterion established was for the student to be 18 years or over and, of 714 academics who responded to the instruments, 77 were excluded due to inconsistencies or blank responses, totaling 637 valid questionnaires. 

To obtain the data a closed instrument was constructed, aiming to assess the demographic and socioeconomic conditions and, regarding the presence of suicidal ideation, the question "In the last 30 days have you thought about killing yourself?" was introduced. The construction of this question was directed by studies that address the theme^10^ ^-^ ^11^. To determine the economic class the Brazil Economic Classification Criterion of the Brazilian Association of Market Research Companies^16^ was used. A second instrument was used to identify alcohol consumption, ASSIST (Alcohol, Smoking and Substance Involvement Screening Test), which aims to detect the risk level of the use of tobacco, alcohol, marijuana, cocaine, stimulants, amphetamine, sedatives, hallucinogens, inhalants, opiates and other drugs. The points obtained indicate the following categories of use: 03 points: occasional use (classified as low-risk consumption), 4-15 points: indicative of abuse (moderate risk), ≥16 points: suggestive of dependence (high risk)^17^. In this study, other psychoactive substances were disregarded, with only the alcohol variable being analyzed. 

The final instrument used was the Major Depression Inventory constructed from the DSM-IV and ICD-10 and used to identify the presence of depressive symptoms. In the Brazilian validated version, the instrument investigates how the person has felt in the previous two weeks, with 10 multiple-choice questions featuring items 8 and 10 with two options. The alternatives range from 0 to 5 (never to all the time). The cutoff equal to or greater than 16 indicates the presence of depressive symptoms^18^.

To allow the collection of data, after authorization of access to the students by the Dean of Undergraduate Studies/UFMT, a schedule was designed for the application of the questionnaires in the class. On the appointed day, a trained applicator explained the aims of the study to the students and delivered the consent form for signature and the questionnaires, which were completed and deposited in boxes placed at the front of the classroom. The confidentiality of responses was ensured through anonymity and the completion of the questionnaire was not compulsory, allowing the student to return it unanswered at any time. Data collection took place in April and May 2015.

The data were compiled in a spreadsheet, using the Microsoft Excel program, and a comparison of the data entered was carried out using the resources of Epi Info version 3.5, with data analysis performed using SPSS version 17.0. A descriptive statistical analysis was conducted, with the presentation of the prevalence, distribution and relative and absolute frequencies data sequentially in order to verify inferential statistical associations between the presence of suicidal ideation (dependent variable) and the other independent variables. 

In this analysis, prevalence ratios were determined and, to test the significance, the chi-square test and when necessary the Fisher exact test were performed, in both cases considering a significance level of 0.05 and confidence interval (CI) of 95%. For the multivariate analysis, a robust Poisson regression model was used and the variables that presented a *p*-value less than 0.20 were tested, with those that presented *p*-values ​​less than 0.05, with 95% CI, remaining in the final model.

The study was approved by the Research Ethics Committee of UFMT under No. 1.021.217, following the ethical principles governing studies with human subjects determined by Resolution No. 466/2012 of the National Health Council (CNS). 

## Results 

The prevalence of suicidal ideation was obtained through the following question: "In the last 30 days have you thought of killing yourself?" A percentage of 9.9% of the students answered yes and 90.1% indicated the no option with CI: 7.68 ; 12.47 and 87.52 ; 92.31, respectively.

Table 1 presents the associations of the demographic, socioeconomic and academic variables with the presence of suicidal ideation. It was observed that the university students included in the lower socioeconomic levels (C1, C2, DE) presented a higher prevalence of suicidal ideation in relation to those classified in the levels, B1 and B2 (PR=1.69, CI:1.01 ; 2.83). Regarding the sexual orientation, it was evidenced that suicidal ideation was significantly associated with the homosexual and bisexual men (*p*=0.008 and *p*<0.001, respectively). Among those without a religious practice, the prevalence rate of suicidal ideation was higher compared to those that reported having a religion (*p*<0.001).

**Table 1:** Association between the demographic, socioeconomic and academic variables of the university students of UFMT, Cuiabá campus, with the presence of suicidal ideation. Cuiabá, MT, Brazil, 2015

Variables		Yes			No		PR**_b_** *	95% CI**^†^**	P-value
		n	(%)		n	(%)			
Age									
	18 to 24 years	48	(10.8)		397	(89.2)	2.40	(0.89; 6.49)	0.068
	25 to 31 years	11	(10.7)		92	(89.3)	2.38	(0.78; 7.20)	0.111
	32 years or more	4	(4.5)		85	(95.5)	1.00	-	-
Gender									
	Female	39	(11.5)		300	(88.5)	1.45	(0.88; 2.32)	0.143
	Male	24	(8.0)		274	(92.0)	1.00	-	-
Marital Status									
	Single	58	(11.0)		485	(89.0)	2.01	(0.83; 4.88)	0.108
	Married	5	(5.0)		89	(95.0)	1.00	-	-
Economic class									
	C1, C2 and D-E	39	(11.8)		292	(88.2)	1.69	(1.01; 2.83)	0.042
	A, B1 and B2	20	(7.0)		267	(93.0)	1.00	-	-
Live alone									
	Yes	12	(11.5)		92	(88.5)	1.19	(0.66; 2.18)	0.538
	No	51	(9.6)		482	(90.4)	1.00	-	-
Sexual orientation									
	Homosexual	10	(20.0)		40	(80.0)	2.54	(1.37; 4.74)	0.008EF**^‡^**
	Bisexual	9	(33.0)		18	(67.0)	4.24	(2.32; 7.76)	<0.001EF
	Heterosexual	44	(7.9)		516	(92.1)	1.00	-	-
Religious practice									
	No	35	(16.3)		180	(83.7)	2.45	(1.54; 3.92)	<0.001
	Yes	28	(6.6)		394	(93.4)	1.00	-	-
Years of the course									
	1**^st^** and 2**^nd^** years	39	(9.4)		374	(90.5)	0.88	(0.54; 1.43)	0.608
	3**^rd^** , 4**^th^** and 5**^th^** years	24	(10.7)		200	(89.2)	1.00	-	-

*PRb: prevalence ratio; †CI: 95% confidence interval; ‡Fisher exact test

Table 2 shows that the university students who reported cases of attempted suicide in the family and among friends were more likely to have suicidal ideation than those who did not report the event (PR=3.15, 95% CI: 1.99 ; 4.99 and PR=1.92, 95% CI: 1.20 ; 3.07, respectively).

**Table 2:** Association between suicidal behavior among friends and families of the university students of UFMT, Cuiabá campus, and the presence of suicidal ideation. Cuiabá, MT, Brazil, 2015.

Variables		Yes			No		PRb*	95% CI**^†^**	P-value
		n	(%)		n	(%)			
Attempted suicide in the family									<0.001
	Yes	26	(22.4)		90	(77.6)	3.15	(1.99; 4.99)	
	No	37	(7.1)		484	(92.9)	1.00	-	-
Suicide in the family									0.118
	Yes	9	(15.8)		48	(84.2)	1.69	(0.88; 3.25)	
	No	54	(9.3)		526	(90.7)	1.00	-	-
Attempted suicide among friends									0.006
	Yes	28	(15.0)		159	(85.0)	1.92	(1.20; 3.07)	
	No	35	(7.8)		415	(92.2)	1.00	-	-
Suicide among friends									0.254
	Yes	11	(13.4)		71	(86.6)	1.42	(0.77; 2.62)	
	No	52	(9.4)		502	(90.6)	1.00	-	-

*PRb: prevalence ratio; †CI: 95% confidence interval

In Table 3, it can be observed that the alcohol consumption and depressive symptoms variables showed statistically significant associations with suicidal ideation, with *p*=0.002 and CI: 1.31 ; 3.34 and *p*<0.001, CI: 5.75 ; 29.9, respectively.

**Table 3:** Prevalence and association of alcohol consumption and depressive symptoms with suicidal ideation among university students of UFMT, Cuiabá campus. Cuiabá, MT, Brazil, 2015

Variables		Yes			No		PRb*	95% CI**^†^**	P-value
		n	(%)		n	(%)			
Alcohol consumption									0.002
	Moderate/high risk	28	(15.9)		148	(84.1)	2.10	(1.31 ; 3.34)	
	Low risk	35	(7.6)		426	(92.4)	1.00	-	-
Presence of depressive symptoms									<0.001
	Yes	57	(21.4)		210	(78.6)	13.3	(5.75 ; 29.9)	
	No	6	(1.4)		363	(98.6)	1.00	-	-

*PRb: prevalence ratio; †CI: 95% confidence interval

Table 4 presents the variables associated with the presence of suicidal ideation after the multiple analysis. The Poisson Regression Model was applied and the variables that remained significant were sexual orientation, in the categories homosexual (*p*=0.009) and bisexual (*p*=0.007), attempted suicide in the family (*p*<0.001) and depressive symptoms (*p*<0.001).

**Table 4:** Factors associated with suicidal ideation in university students of UFMT, Cuiabá campus. Cuiabá, MT, Brazil, 2015.

Variables	PRa*	95% CI**^†^**	P-value
Sexual orientation (homosexual)	2.05	(1.20 ; 3.52)	0.009
Sexual orientation (bisexual)	2.37	(1.27 ; 4.44)	0.007
Suicide attempt in the family	2.30	(1.48 ; 3.58)	<0.001
Depressive symptoms	11.00	(4.71 ; 25.70)	<0.001

*Adjusted prevalence ratio; †Confidence Interval

In table 4 it can be seen that presence of depressive symptoms was the variable that showed a marked association with suicidal ideation in the previous 30 days, followed by sexual orientation (bisexual), attempted suicide in the family and sexual orientation (homosexual).

## Discussion 

The present study showed a prevalence of 9.9% (n=63) of suicidal ideation in the previous 30 days among university students. A similar study carried out in a private university in northern Portugal, with 366 students, found that 12.6% had thought about suicide in the life time, 10.7% in the previous year and 1.1% in previous weeks^1^. Lower prevalences were found in a comparative study conducted with medical students in Austria (n=320) and Turkey (n = 326), where 5% of the Austrian university students and 3.7% of the Turkish students had thought about suicide in the previous weeks^12^. The different prevalences obtained may have occurred due to the different types of instruments used to determine the phenomenon, because of specific features and conditions in the different regions and countries, or due to the time factor, i.e. the period in which the participants reported the presence of ideation, which reinforces the need to explore the theme in the literature more consistently.

It should be emphasized, considering these prevalences, that the nurse has skills that can help in the management of the presence of suicidal ideation. Thus, this professional may be a key element in groups that promote actions directed toward this theme, as well as in the composition of management teams to discuss policies to minimize the phenomenon in the university environment.

Among the demographic and economic variables, the economic class was associated with suicidal ideation among the students investigated, where those (n=39 of 331) belonging to the class C1, C2 and D-E presented 1.69 times more suicidal ideation compared to students in the higher economic classification levels (A, B1 and B2). A similar study with 101 university students of Massachusetts/USA, despite being a developed country, found that 6.6% (n = 19) fell into the group of low per capita income of the family^15^. Also, a study performed in a public university of São Paulo, encountered different findings in its population in relation to those evidenced in this study, with 53.1% of the students surveyed belonging to economic class A^19^.

In relation to sexual orientation and its association with suicidal ideation shown in the bivariate analysis, the students who reported being homosexual or bisexual presented more suicidal ideation (PR=2.5, and 4.24, respectively) compared to those that reported being heterosexual. In a study conducted in the USA with university students (n=1,085), this finding was confirmed, with a prevalence ratio of 4.7 that reported being homosexual or bisexual in relation to the heterosexuals. In the adjusted analysis of the present study this variable retained a strong association (*p*=0.007) for the bisexual students. Similarly, these categories remained in the model after the regression^2^.

The condition of heterosexuality, socially, is configured as a major reference regarding the desires, ideals, principles and values ​​emerging, thus there is the feeling of superiority in relation to all the other various expressions of sexuality, causing those that do not follow this reference to feel excluded and different^13^. This condition, as a choice of a sexual orientation other than that socially expected, can lead to several consequences among university students who define themselves as homosexual and bisexual, as being the target of prejudice can arouse immense suffering and intense emotional fragility, leading to the production of suicidal ideation^20^.

Regarding the religious practice variable, the association between the not practicing a religion and the presence of suicidal ideation evidenced here was also found in a study with students from a University of Florida- USA^3^. This association, although it did not remain in the adjusted model may suggest that having a religious practice contributes to the spiritual welfare of the student inhibiting the emergence of suicidal ideation. 

The exercise of religious practices, such as prayer, meditation and other manifestations of belief, contribute to the balance of emotions and feelings^21^. Thus, having a religious practice is configured as a protective factor for the individual and for the emergence of suicidal ideation. Through this context, which involves cultural aspects and subjective values​​, it is important for the scientific literature to investigate religious practice (belief/habits) and its possible relationship with suicidal ideation among university students^3^, with future studies focused on this still little explored theme. 

Regarding suicide attempts in the family and among friends, these variables were associated with suicidal ideation. A study with a public in an age group slightly lower than in the sample of the present study (students aged 15 to 19 years) showed that the young people that had a friend who had attempted suicide were twice as likely to present suicidal ideation compared to those who did not know anyone who had attempted suicide. Furthermore, according to the study, of the 188 young people who had suicidal ideation, 24% had someone in the family who had attempted suicide^9^.

The suicide attempt in the family variable was found to be strongly associated with suicidal ideation when the final model was applied (*p*<0.001). Interpersonal relationships can exert a strong influence on the behavior of an individual. Therefore, being involved with someone who has already made a suicide attempt can lead to behavior of the reproduction of the act, becoming a behavior learned as a way of resolving conflicts, thereby increasing cases of suicide^8^. 

Regarding the use of alcohol, students who showed high/moderate risk for this use were twice as likely to present suicidal ideation compared to the students of the low-risk category. In a study of 1,100 University students in the USA, using a screening test for alcohol use (AUDIT), the authors demonstrated a significant association between heavy alcohol consumption and suicidal ideation (*p*=0.001)^22^. A study that tracked alcohol consumption in students, performed in a public higher education institution, found that of 32 university students, 28 presented risk use of this substance^19^.

Upon entering academia, the university student is faced with a new environment that brings the possibility of socialization through parties, relaxation of parental control and accountability for alcohol use, which may promote increased use in this population. It should be mentioned that alcohol consumption among university students has been associated with the presence of suicidal ideation and attempts^23^. In the present study this variable, although not remaining in the final model, indicates the need for a thorough look at this population, as in this stage they are more vulnerable to the initiation and constant consumption of alcohol, as well as its highest incidence^24^.

The association between the presence of depressive symptoms and suicidal ideation is highlighted, because among the students who had these symptoms (*n*=267), 21.4% had thought of killing themselves in the previous 30 days. This was the variable most strongly associated with the presence of suicidal ideation among the students. An investigation conducted in the USA, with 2,843 university students showed a prevalence of 2% of suicidal ideation in this population during the academic career and, among these, 17% revealed the presence of depressive symptoms and 9% had a diagnosis of depression^22^.

In the multivariate analysis this variable remained associated with suicidal ideation and demonstrated a high prevalence ratio. Individuals living with psychological distress and depressive symptoms often externalize the desire to die/kill themselves, reasoning that committing suicide is the solution, carrying it out effectively^9^. Thus, suicide emerges as the only existing way out faced with a current confrontational moment and negative expectations for the future^14^. 

The negative aspects that arise when a person has depressive symptoms can lead to a lack of meaning in life and a sense of powerlessness, with the emergence of these feelings strengthened in this context, predisposing the student to suicidal ideation^6^. It should be noted that even though depressive symptoms have been described as a factor associated with suicidal ideation, there can be no affirmation of a possible cause and effect relationship between depressive symptoms and suicidal ideation, as many people with depressive symptoms do not necessarily wish to end their lives^15^.

The factors associated with suicidal ideation among university students, investigated in this study, have also been evidenced in the general population^4^ ^,^ ^20^. The consequences of suicide may involve social, economic and emotional harm to family and friends, especially in younger age groups, where the impact may be more pronounced^20^. Given this context, the need is highlighted for nurses to hone their skills, from the assessment to the implementation of care in the different areas in which they operate, significantly contributing to addressing this growing public health problem. 

The study presents, like any research, advantages and some limitations that need to be highlighted. The advantage referred to is that this subject is still little explored in the literature, especially in the young population, and specifically in university students, considering the increase in suicide rates in this populational group. 

Regarding the limitations, the depression scale used for the assessment of depressive symptoms does not have cutoff points adapted to the university population, therefore the findings should be viewed with caution, with the need for further studies to provide more secure analysis of levels of depressive symptoms in students. Another aspect is related to the fact that ideation is a very subjective phenomenon that occurs under the influence of different factors that have not been analyzed here and would require further studies. Finally the cross-sectional design can be a limitation, since it cannot determine the temporality of the factors found.

## Conclusion 

It was found that that the variables associated with suicidal ideation were economic class, sexual orientation, religious practice, suicide attempts in the family and among friends, moderate and high risk alcohol consumption and depressive symptoms. However, in the multivariate analysis, sexual orientation, suicide attempts in the family and depressive symptoms remained in the adjusted model.

These findings constitute a situational diagnosis that higher can assist education institutions in the promotion of prevention and coping actions regarding these questions, as well as help health professionals, who work within the campus or attend students off site, to understand the importance of measures that identify and minimize the situation.
